# Nutrients Utilization in Obese Individuals with and without Hypertriglyceridemia

**DOI:** 10.3390/nu6020790

**Published:** 2014-02-21

**Authors:** Tiziana Montalcini, Theodora Lamprinoudi, Attilio Morrone, Elisa Mazza, Carmine Gazzaruso, Stefano Romeo, Arturo Pujia

**Affiliations:** 1 Clinical Nutrition Unit, Department of Medical and Surgical Science, University Magna Grecia, Catanzaro 88100, Italy; E-Mails: tlamprinoudi@hotmail.it (T.L.); attiliom88@gmail.com (A.M.); elisamazza@inwind.it (E.M.); romeo@unicz.it (S.R.); pujia@unicz.it (A.P.); 2 Diabetes, Endocrine-metabolic Dis. Cardiovasc. Prevention Unit, Clinical Inst. “Beato Matteo”, Department of Internal Medicine, I.R.C.C.S. Policlinico San Donato Milanese, Vigevano 27029, Italy; E-Mail: c.gazzaruso@gmail.com; 3 Department of Molecular and Clinical Medicine, Sahlgrenska Center for Cardiovascolar and Metabolic Research, University of Gothenburg, Göteborg 40330, Sweden

**Keywords:** triglyceride, fatty acid, obesity, fatty acid oxidation, lipids, nutrients, indirect calorimetry, carboidrates, respiratory quotient

## Abstract

Background: Low fat utilization is linked to weight gain and to the presence of certain atherosclerosis markers. It is not clear whether the presence of hyperlipidemia can further affect nutrients utilization. The main objective of this study was to investigate the fasting fuel utilization of obese subjects suffering from hypertriglyceridemia, and to compare it with that of individuals that are solely obese. Method: We recruited 20 obese individuals with hypertriglyceridemia and 20 matched individuals not affected by hypertriglyceridemia. The fuel utilization (respiratory quotient) was measured by respiratory gas exchange, by Indirect Calorimetry. Results: There was a significant difference in fuel utilization and HDL-cholesterol between cases and controls (respiratory quotient 0.89 ± 0.07 *vs*. 0.84 ± 0.06; *p* = 0.020 respectively). The univariate and multivariate linear regression analysis confirmed that hypertrygliceridemia was positively correlated to the respiratory quotient (*p* = 0.035). Conclusion: obese subjects with hypertriglyceridemia had a higher respiratory quotient in comparison to unaffected subjects. This could suggest a limitation in the beta-oxidation mechanisms; this could actually imply that fatty acids may be redirected from oxidation to reesterification into triglycerides. The study could suggest the presence of different mechanisms unrelated to obesity and also a potential new therapeutic target for hypertriglyceridemia management.

## 1. Introduction

It is well known that fatty acids (FA) are the primary fuel for several tissues and organs like resting muscle, liver and heart [1,2,3] and that an increased supply of FA inhibits glucose utilization [4,5]. Furthermore, it is widely known that after an overnight fast, subjects receiving a balanced diet tend to burn fat as main substrate [6]. The importance of assess the fuel utilization of a subject lies in the demonstrated high rate of weight gain [7] and the presence of certain predictors of cardiovascular diseases [8,9] in individuals with a low fat utilization. Much remains to be clarified about the condition of excess free FA flux in already obese individuals. Excess of free FA as well as lipid mobilization are considered important factors in increased hepatic very-low-density lipoprotein (VLDL)—triglyceride (TG) secretion [10]. Both increased synthesis and/or decreased clearance of the VLDL lead to high circulating TG concentration. The elevated TG level is an independent risk factor for cardiovascular disease (CVD) [11,12,13]. In the treatment of hypertriglyceridemia and combined hyperlipoproteinemia, life-style changes play a key role. Therefore, the aim of this study was to compare the fasting fuel utilization of obese subjects affected by hypertriglyceridemia with unaffected individuals that are solely obese, and to investigate the relationship between the index of nutrient utilization, the Respiratory Quotient (RQ), and triglycerides.

## 2. Method

In this case-control study we recruited 40 unrelated obese individuals; 20 affected by hypertriglyceridemia (cases) and 20 unaffected (controls), matched by age, body mass index (BMI) and gender. The hypertriglyceridemic patients were blank subjects, ascertained through our Lipid Clinic at the Clinical Nutrition Unit in the year 2013. For the purposes of this investigation, based on previous studies [14,15,16], participants were classified according to TG levels and were referred to the controls (if TG was lower than 200 mg/dL) or cases (TG equal or greater than 250 mg/dL) group. The population included both gender and age 25–70 years old. We enrolled subjects who were following a nutritionally balanced diet (*i.e.*, a diet that supplied 50%–55% of calories from carbohydrates, 25%–30% from fat, and 18%–20% from protein) based on a nutritional intake assessment. Additional cases were recruited if hyperlipidemia was present in at least one first degree relative. We excluded individuals with diabetes, alcohol abuse, pregnancy, or taking corticosteroids, oral estrogen, tamoxifen and thiazides. We also excluded subjects taking anti-obesity medications, psychotropic drugs and chronotropic agents, with clinical evidence of debilitating diseases, like chronic illness (cancer, renal failure, liver insufficiency and chronic obstructive pulmonary disease) and thyroid dysfunction.

The following criteria were used to define the distinct cardio-metabolic risk factors; diabetes: fasting blood glucose ≥ 126 mg/dL or antidiabetic treatment; familiar hyperlipidemia: total cholesterol > 200 mg/dL and/or triglycerides > 250 mg/dL or lipid lowering drugs use in at least a first degree relative; hypertension: systolic blood pressure ≥140 mmHg and/or diastolic blood pressure ≥ 90 mmHg or antihypertensive treatment; obesity: BMI ≥ 30 kg/m^2^ [17,18].

All tests were performed after a 12 h overnight fasting. Before tests, we gave no particular suggestions on the menu for the meal, but we requested that the dinner before the experiments would have to include types of foods and drinks usually consumed. Indeed they have no caffeinated beverages between their evening meal and the conclusion of the tests on the examination’s morning. Written informed consent was obtained. The protocol was approved by local ethical committee at the University Hospital (projects codes 2013-1/CE). The investigation conforms to the principles outlined in the Declaration of Helsinki. 

### 2.1. Nutritional Intake and Anthropometric Measurements

The participant’s nutritional intake was calculated using the nutritional software MetaDieta 3.0.1 (Metedasrl, S. Benedetto del Tronto, Italy). Body weight was measured before breakfast with the subjects lightly dressed, subtracting the weight of clothes. Body weight was measured with a calibrated scale and height measured with a wall-mounted stadiometer. BMI was calculated with the following equation: weight (kg)/(height (m))^2^. Waist and hip circumferences (WC and HC) were measured with a nonstretchable tape over the unclothed abdomen at the narrowest point between the costal margin and iliac crest and over light clothing at the level of the widest diameter around the buttocks, respectively, as described in the past [19].

### 2.2. Blood Pressure Measurement

The measurement of the systemic BP of both arms was obtained by a mercury sphygmomanometer (systolic blood pressure—SBP and diastolic blood pressure—DBP) as previously described [9,20]. Clinic BP was obtained in the supine patients, after 5 min of quiet rest. A minimum of three BP readings was taken using an appropriate BP cuff size (the inflatable part of the BP cuff covered about 80% of the circumference of upper arm).

### 2.3. RQ and RMR Measurement

Fasting RQ and resting metabolic rate (RMR) were measured with the participants in their postabsorptive state in a sedentary position. Respiratory gas exchange was measured by Indirect Calorimetry using the open circuit technique between the hours of 7:00 am and 8:30 am after 48-h abstention from exercise. The Indirect Calorimetry instrument (Viasys Healthcare, Hoechberg, Germany) was used for all measurements. The participant rested quietly for 30 min in an isolated room with temperature controlled (21–24 °C) environment. The subject was then placed in a ventilated hood for at least 30 min, until steady state was achieved. Criteria for a valid measurement was a minimum of 15 min of steady state, with steady state determined as less than 10% fluctuation in minute ventilation and oxygen consumption and less than 5% fluctuation in RQ. RQ was calculated as CO_2_ production/O_2_ consumption [9,21].

### 2.4. Biochemical Evaluation

Venous blood was collected after fasting overnight into vacutainer tubes (Becton & Dickinson) and centrifuged within 4 h. Serum glucose, creatinine, total cholesterol, high density lipoprotein (HDL)-cholesterol, triglycerides, uric acid, insulin were measured with Enzymatic colorimetric test. ApoB100 was measured with nephelemetric method. Homa index was calculated with the following formula: [glucose (mmol/L) × insulin (mU/L)]/22.5. We used fasting lipid levels to calculate the value for low density lipoprotein (LDL) cholesterol (Friedewald formula). Quality control was assessed daily for all determinations.

### 2.5. Statistical Analysis

The data is reported as average ± S.D. The *t*-test was used to compare the averages between cases and controls. The univariate analysis was used to determine all the factors correlated to the RQ. The stepwise multivariate linear regression analysis was used to test for confounding variables. In particular, the continuous and categorical variables included in this analysis were those correlated to the RQ (dependent variable) in univariate analysis with a *p* < 0.1. Significant differences were expected to be found at *p* < 0.05. All comparisons were performed using the SPSS 20.0 for Windows (Chicago, IL, USA).

## 3. Results

**Figure 1:**
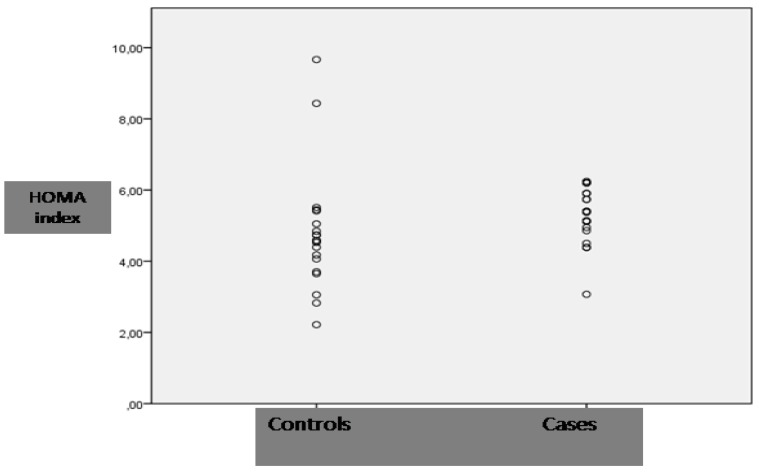
Depicts the scatter plot of individual HOMA index of cases and controls.

The average age was 49.3 ± 8 years, for cases and 49.7 ± 9 years, for controls (*p* = *ns* between groups). 11 Subjects in each group were male. The characteristics of the population, relative to the presence of hypertriglyceridemia, are showed in Table 1. There was a significant difference in the RQ and HDL-cholesterol between cases and controls (0.89 ± 0.07 *vs.* 0.84 ± 0.06; *p* = 0.020 for RQ; Table 1). Among the variables included in the univariate analysis (continuous variables: age, SBP, DBP, BMI, WC, HC, RMR, glucose, total cholesterol, triglycerides, HDL-cholesterol, apo B 100, creatinin, uric acid, insulin; categorical: “hypertriglyceridemia”), both the triglycerides and the condition of hypertriglyceridemia were correlated to RQ (*r* = 0.31; *p* = 0.050 and *r* = 0.36; *p* = 0.020 respectively). Additional HDL-cholesterol and triglycerides were correlated (*r* = −0.58; *p* < 0.001). The multivariate linear regression analysis, including only these two variables, confirmed that the presence of hypertrygliceridemia was positively correlated to RQ (*p* = 0.035; Table 2). We performed the scatter plot of individual Homeostatic Model Assessment (HOMA) Page: 4 index in both cases and controls (Figure 1).

**Table 1:** Characteristics of the population according to TG levels.

Variables	Controls	Cases	*p*
Age (years)	49.7 ± 9	49.3 ± 8	0.901
RMR (Joule)	6970.18 ± 1105	7223.06 ± 1419	0.534
RQ	0.84 ± 0.06	0.89 ± 0.07	0.020
BMI	36.3 ± 5	35.7 ± 5	0.782
Weight (kg)	97.8 ± 19	95.6 ± 23	0.749
WC (cm)	111.3 ± 13	116.1 ± 19	0.406
HC (cm)	115.1 ± 11	114.7 ± 15	0.931
SBP (mmHg)	126.5 ± 16	127.6 ± 16	0.831
DBP (mmHg)	78.4 ± 7	80.0 ± 10	0.586
Glucose (mmol/L)	91 ± 10	93.7 ± 9	0.398
Creatinin (µmol/L)	0.82 ± 0.2	0.77 ± 0.2	0.407
Tot Cholesterol (mmol/L)	205.6 ± 43	220.6 ± 34	0.235
LDL-Cholesterol (mmol/L)	135.1 ± 40	125.4 ± 36	0.467
HDL-Cholesterol (mmol/L)	49.15 ± 12	37.61 ± 9	0.002
Triglyceride (mmol/L)	109.1 ± 33	341.5 ± 125	<0.001
Uric Acid (mmol/L)	5.1 ± 1.0	6.1 ± 1	0.070
Apo B100 (mmol/L)	1.15 ± 0.3	1.20 ± 0.2	0.691
Insulin (pmol/L)	148.8 ± 71	161.8 ± 28	0.712
HOMA index	4.7 ± 1.7	5.2 ± 0.8	0.233

**Table 2:** Multivariate linear regression analisys—dependent variable RQ.

Independent Variables	B	SE	Beta	*t*	*p*
Hypertriglyceridemia	0.052	0.024	0.343	2.192	0.035

## 4. Discussion

In our investigation, we found a significant difference in the fuel utilization of obese subjects with a high level of serum triglycerides in comparison to those with a normal level, matched by age and gender (Table 1). In particular, subjects with hypertriglyceridemia had a higher RQ (suggesting high carbohydrate and low fat utilization) than the control subjects. In addition, in the univariate analysis we found a positive association between RQ and triglycerides, and in the multivariate analysis there was a positive relation between RQ and the condition of hypertriglyceridemia (Table 2). 

This is an unprecedented finding, never investigated to date. Limited research has been conducted studying the substrate utilization in patients with hypertriglyceridemia. A greater understanding of this issue may contribute to providing appropriate nutritional advice to these subjects, which have an increased susceptibility to CVD. Furthermore, the concepts emerging from our work may add intriguing and new information relative to the mechanisms involved in obesity associated to hypertriglyceridemia. In fact, it is important to note that in our study, the average RMR, BMI, WC, HC, glucose and total cholesterol was not different between cases and controls, so these variables cannot account for the difference in RQ between the two groups. Consequently, although our study was not designed to explore the underling mechanisms, some clarifications are needed. It is known that after an overnight fast, a subject having a balanced diet mainly burns fats [6]. In this work, we showed that subjects having high serum triglycerides seem to not be able to utilize them at fast. Therefore, they may need to sustain high rates of carbohydrate oxidation to compensate for the inability to use fat as a fuel, as showed by the high RQ. In contrast with our finding, it has been shown that hypertriglyceridemia is associated with impaired free FA suppression, high FA levels [7,8,9,10,21,22,23,24,25], insulin resistance [23] and inhibition of carbohydrate oxidation [26]. However, our data is in line with a recent investigation [27]. This study showed how the RQ increased during the combined administration of insulin/glucose together with fat, which is a condition similar to hypertriglyceridemia [27]. In particular, in this study the authors showed that the administration of fat during the hypertriglyceridemic clamp did not change RQ, while during the combined administration of insulin/glucose together with fat the RQ increased [27]. Under these conditions they hypothesized the increased channeling of FFA toward triglycerides synthesis rather than oxidation, along with a significant increase in carbohydrate oxidation [27]. Insulin may play a role in suppressing the lipid oxidation. Thus, in individuals with both insulin sensitivity and hypertriglyceridemia, the excess of lipids seems to be stored, lipid oxidation inhibited and RQ increased. In insulin-resistance condition, the excess of lipids seems to be associated with the increased lipid oxidation and RQ. In our population, both serum glucose and insulin were higher in the cases than in the controls but the difference was not statistically significant, confirming these mechanisms. However, at this moment, the mechanisms involved are not fully clarified [28,29,30,31]. We hypothesized that RQ assessment may represent an attractive identification tool for individuals with obesity at risk of diabetes, and/or those that may need a particular diet and/or those that may be responsive to certain medications [7,8,9,23,24]. In this regard, it has been shown that carnitine supplementation can increase the lipid utilization by muscle and, most importantly, can reduce RQ during exercise [24]. Furthermore, these results might help to identify innovative potential therapeutic targets.

The small sample size may be a limitation of our study, however despite this, the effects based on size were large.

## 5. Conclusions

In conclusion, RQ assessment in obese individuals may be useful to complete their phenotypic characterization and it may help to identify individuals needing special therapeutic strategies, such as those with hypertriglyceridemia.

## References

[1] Bjorkman O. (1986). Fuel metabolism during exercise in normal and diabetic man. Diabetes Metab. Rev..

[2] Wisneski J.A., Gertz E.W., Neese R.A., Mayr M. (1987). Myocardial metabolism of free fatty acids. Studies with 14C-labeled substrates in humans. J. Clin. Investig..

[3] Gold M., Spitzer J.J. (1964). Metabolism of free fatty acids by myocardium and kidney. Am. J. Physiol..

[4] Andres R., Cader G., Zierler K.L. (1956). The quantitatively minor role of carbohydrate in oxidative metabolism by skeletal muscle in intact man in the basal state: Measurements of oxygen and glucose uptake and carbon dioxide and lactate production in the forearm. J. Clin. Investig..

[5] Ferrannini E., Barrett E.J., Bevilacqua S., DeFronzo R.A. (1983). Effect of fatty acids on glucose production and utilization in man. J. Clin. Investig..

[6] Mcneill G., Bruce A.C., Ralph A., James W.P.T. (1988). Interindividual differences in fasting nutrient oxidation and the influence of diet composition. Int. J. Obes..

[7] Zurlo F., Lillioja S., Esposito-Del Puente A., Nyomba B.L., Raz I., Saad M.F., Swinburn B.A., Knowler W.C., Bogardus C., Ravussin E. (1990). Low ratio of fat to carbohydrate oxidation as predictor of weight gain: Study of 24-h RQ. Am. J. Physiol. Endocrinol. Metab..

[8] Montalcini T., Gazzaruso C., Ferro Y., Migliaccio V., Rotundo S., Castagna A., Montalcini T., Gazzaruso C., Ferro Y., Migliaccio V. (2012). Metabolic fuel utilization and subclinical atherosclerosis in overweight/obese subjects. Endocrine.

[9] Ferro Y., Gazzaruso C., Coppola A., Romeo S., Migliaccio V., Giustina A., Ferro Y., Gazzaruso C., Coppola A., Romeo S. (2013). Fat utilization and arterial hypertension in overweight/obese subjects. J. Transl. Med..

[10] Kissebah A.H., Alfarsi S., Adams P.W., Seed M., Folkyard J., Wynn V. (1976). Transport kinetics of plasma free fatty acid, very low density lipoprotein triglycerides and apoprotein in patients with endogenous hypertriglyceridaemia. Atherosclerosis.

[11] Sarwar N., Danesh J., Eiriksdottir G., Sigurdsson G., Wareham N., Bingham S., Sarwar N., Danesh J., Eiriksdottir G., Sigurdsson G. (2007). Triglycerides and the risk of coronary heart disease: 10,158 incident cases among 262,525 participants in 29 Western prospective studies. Circulation.

[12] Hokanson J.E., Austin M.A. (1996). Plasma triglyceride level is a risk factor for cardiovascular disease independent of high-density lipoprotein cholesterol level: A meta-analysis of population-based prospective studies. J. Cardiovasc. Risk.

[13] Carey V.J., Bishop L., Laranjo N., Harshfield B.J., Kwiat C., Sacks F.M. (2010). Contribution of high plasma triglycerides and low high-density lipoprotein cholesterol to residual risk of coronary heart disease after establishment of low-density lipoprotein cholesterol control. Am. J. Cardiol..

[14] Stein E.A., Lane M., Laskarzewski P. (1998). Comparison of statins in hypertriglyceridemia. Am. J. Cardiol..

[15] Llurba E., Casals E., Domínguez C., Delgado J., Mercadé I., Crispi F., Llurba E., Casals E., Domínguez C., Delgado J. (2005). Atherogenic lipoprotein subfraction profile in preeclamptic women with and without high triglycerides: Different pathophysiologic subsets in preeclampsia. Metabolism.

[16] Damci T., Tatliagac S., Osar Z., Ilkova H. (2003). Fenofibrate treatment is associated with better glycemic control and lower serum leptin and insulin levels in type 2 diabetic patients with hypertriglyceridemia. Eur. J. Intern. Med..

[17] National High Blood Pressure Education Program Working Group (1994). National High Blood Pressure Education Program Working Group Report on hypertension in the elderly. Hypertension.

[18] Psaty B.M., Furberg C.D., Kuller L.H., Bild D.E., Rautaharju P.M., Polak J.F., Psaty B.M., Furberg C.D., Kuller L.H., Bild D.E. (1999). Traditional risk factors and subclinical disease measures as predictors of first myocardial infarction in older adults: The Cardiovascular Health Study. Arch. Intern. Med..

[19] Montalcini T., Gorgone G., Garzaniti A., Gazzaruso C., Pujia A. (2010). Artery remodeling and abdominal adiposity in nonobese postmenopausal women. Eur. J. Clin. Nutr..

[20] Montalcini T., Gorgone G., Fava A., Romeo S., Gazzaruso C., Pujia A. (2012). Carotid and brachial arterial enlargement in postmenopausal women with hypertension. Menopause.

[21] Zemel M.B., Bruckbauer A. (2012). Effects of a leucine and pyridoxine-containing nutraceutical on fat oxidation, and oxidative and inflammatory stress in overweight and obese subjects. Nutrients.

[22] Byrne C.D., Wareham N.J., Brown D.C., Clark P.M., Cox L.J., Day N.E., Byrne C.D., Wareham N.J., Brown D.C., Clark P.M. (1994). Hypertriglyceridaemia in subjects with normal and abnormal glucose tolerance: Relative contributions of insulin secretion, insulin resistance and suppression of plasma non-esterified fatty acids. Diabetologia.

[23] Roden M., Price T.B., Perseghin G., Petersen K.F., Rothman D.L., Cline G.W., Roden M., Price T.B., Perseghin G., Petersen K.F. (1996). Mechanism of free fatty acid-induced insulin resistance in humans. J. Clin. Investig..

[24] Gorostiaga E.M., Maurer C.A., Eclache J.P. (1989). Decrease in respiratory quotient during exercise following l-carnitine supplementation. Int. J. Sports Med..

[25] Somesh B.P., Verma M.K., Sadasivuni M.K., Mammen-Oommen A., Biswas S., Shilpa P.C., Reddy A.K., Yateesh A.N., Pallavi P.M., Nethra S. (2013). Chronic glucolipotoxic conditions in pancreatic islets impair insulin secretion due to dysregulated calcium dynamics, glucose responsiveness and mitochondrial activity. BMC Cell Biol..

[26] Boden G., Jadali F., White J., Liang Y., Mozzoli M., Chen X., Boden G., Jadali F., White J., Liang Y. (1991). Effects of fat on insulin-stimulated carbohydrate metabolism in normal men. J. Clin. Investig..

[27] Åberg V., Thörne A., Alvestrand A., Nordenström J. (2012). Combined hypertriglyceridemic and insulin-glucose clamps for the characterization of substrate oxidation and plasma elimination of a long-chain triglyceride emulsion in healthy men. Metabolism.

[28] Bonen A., Parolin M.L., Steinberg G.R., Calles-Escandon J., Tandon N.N., Glatz J.F., Bonen A., Parolin M.L., Steinberg G.R., Calles-Escandon J. (2004). Triacylglycerol accumulation in human obesity and type 2 diabetes is associated with increased rates of skeletal muscle fatty acid transport and increased sarcolemmal FAT/CD36. FASEB J..

[29] Steensberg A., Keller C., Starkie R.L., Osada T., Febbraio M.A., Pedersen B.K. (2002). IL-6 and TNF-alpha expression in, and release from, contracting human skeletal muscle. Am. J. Physiol. Endocrinol. Metab..

[30] Chabowski A., Zmijewska M., Gorski J., Bonen A., Kaminski K., Kozuch M., Chabowski A., Zmijewska M., Gorski J., Bonen A. (2008). IL-6 deficiency increases fatty acid transporters and intramuscular lipid contentin red but not white skeletal muscle. J. Physiol. Pharmacol..

[31] Nyman L.R., Tian L., Hamm D.A., Schoeb T.R., Gower B.A., Nagy T.R., Nyman L.R., Wood P.A. (2011). Long term effects of high fat or high carbohydrate diets on glucose tolerance in mice with heterozygous carnitine palmitoyltransferase-1a (CPT-1a) deficiency: Diet influences on CPT1a deficient mice. Nutr. Diabetes.

